# Giant paratesticular spindle cell lipoma: A rare case report

**DOI:** 10.1016/j.ijscr.2024.110016

**Published:** 2024-07-06

**Authors:** Putu Kurnia Darma Pratama, Tetuka Bagus Laksita, Karinda Triharyu Caesari Putri, Lukman Hakim

**Affiliations:** a Department of Urology, Faculty of Medicine, Universitas Airlangga, Surabaya, Indonesia; b Department of Urology, Dr. Soetomo General Academic Hospital, Surabaya, Indonesia; c Department of Urology, Prof. Dr. Margono Soekarjo, Purwokerto, Indonesia; d Universitas Airlangga Teaching Hospital, Surabaya, Indonesia

**Keywords:** Scrotal mass, Testicular mass, Paratesticular spindle cell lipoma, A rare case, Case report

## Abstract

**Introduction:**

Spindle cell lipoma (SCL) is a rare condition primarily occurring in subcutaneous tissue. Only 4 cases of paratesticular SCL have been reported.

**Case presentation:**

A 51-year-old man presented with a painless mass in his left testicle that has grown for nine years. Physical examination revealed a soft, smooth-surfaced left scrotal mass measuring 30 × 30 cm, which did not transilluminate. A contrast-enhanced abdominal-pelvic CT scan showed a paratesticular mass measuring 31.1 × 15.1 × 30.5 cm extending to the spermatic cord. Preoperative tumor markers, including Alpha-Fetoprotein (AFP), Human Chorionic Gonadotropin (HCG), and Lactate Dehydrogenase (LDH), were within normal ranges. Surgical exploration and excision successfully removed the tumor, measuring 39.0 × 37.0 × 16.0 cm and weighing 10 kg, revealing a spindle cell lipoma on pathology examination. Immunohistochemistry testing for CD34 was positive. Three months post-surgery, the patient was in good health with normal sexual function.

**Discussion:**

Paratesticular SCL is a benign neoplasm. Distinguishing spindle cell lipoma from liposarcoma radiologically is challenging. Therefore, a biopsy and histopathological examination are essential. CD34 Immunohistochemistry aids in determining SCL from liposarcoma. Complete excision following thorough preoperative preparation and accurate diagnostic procedures is recommended.

**Conclusion:**

Paratesticular SCL cases are rare, with good preoperative preparation and accurate post-operative diagnosis; good results will likely be expected, and this case report will likely contribute to ongoing research to enhance the understanding and management of paratesticular SCL cases.

## Highlights

• Only four cases of paratesticular spindle cell lipoma (SCL), which primarily develops in the subcutaneous tissue, have been explained. stating more that our case is a rare case report.
• Testicular lumps can have various causes, and differential diagnoses are possible, one possible differential diagnosis is Spindle Cell Lymphoma (SCL), although this condition is rare.
• Spindle cell lipoma and liposarcoma are difficult to recognize radiologically, a biopsy and histological evaluation are necessary.
• It is expected that paratesticular SCL cases will have satisfactory outcomes, without recurrence, with appropriate preoperative planning and postoperative diagnosis.

## 1 Introduction

Spindle cell lipoma (SCL) is a benign tumor characterized by the replacement of mature adipose cells with collagen tissue, forming spindle cells. It typically develops in the subcutaneous tissue of the upper back, back of the neck, and shoulders in men aged 40–70 years [1,2]. Incidences in other organs are exceedingly rare, accounting for only 1.5 % of all cases, with just four reported cases of paratesticular SCL [3,4].

Paratesticular SCL manifests as a painless enlarging mass in the scrotum. Upon physical examination, the mass is soft and localized unilaterally within the scrotum [3,5]. Clinically, paratesticular SCL can mimic an inguinal hernia or testicular malignancy. Previous cases have involved laboratory tests for tumor markers like alpha-fetoprotein (AFP), beta human chorionic gonadotropin (bHCG), and lactate dehydrogenase (LDH). Radiological assessments, such as contrast-enhanced computed tomography (CT) or magnetic resonance imaging (MRI), are crucial for confirming the diagnosis [1,3,5].

Treatment for paratesticular SCL typically involves total excision of the tumor as curative therapy. Differential diagnosis considerations include liposarcoma, known for its high recurrence rate (30–50 % within five years) [6]. Adequate preoperative preparation and precise post-operative diagnosis are vital to prevent diagnostic errors and ensure favorable outcomes in SCL cases. This case report will delve into the diagnostic and surgical management of paratesticular SCL, a rare condition, following the SCARE Guideline checklist. [7]

## 2 Case presentation

**Fig. 1:**
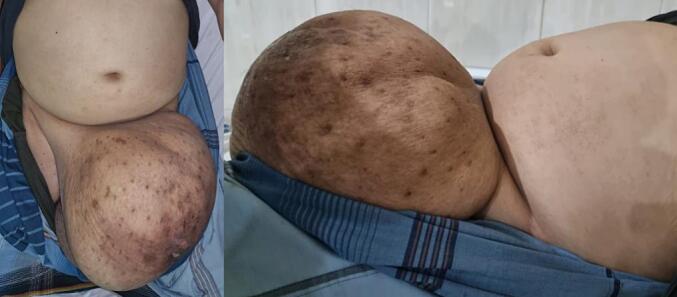
Clinical features of paratesticular mass.

A 51-year-old married man with two children presented at the urology clinic with a chief complaint of a mass in his left testicle that has grown for nine years, The size progressively increased without causing pain. Over the past five years, the mass had enlarged significantly, leading to considerable discomfort because it has obstructed the patient's genitals during urination and sexual activity. Despite these symptoms, the patient avoided medical treatment due to embarrassment and apprehension about surgical intervention. He denied any history of trauma, weight loss, or family members with similar complaints. A scrotal mass measured 30 × 30 cm was identified during the physical examination. It displayed a soft consistency and smooth surface with no transillumination. The assessment of the testicles and penis was challenging due to obstruction by the scrotal mass (Fig. 1).

**Fig. 2:**
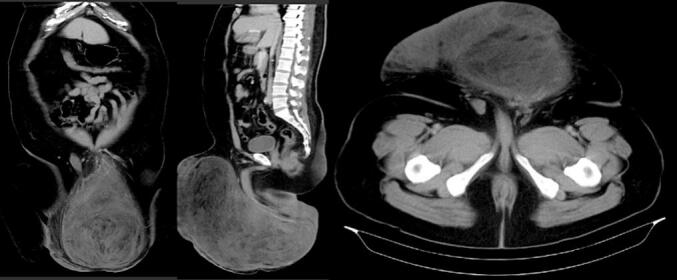
Abdomen-Pelvis CT-Scan with Contrast.

The diagnostic results of a complete blood count, tumor markers like AFP, HCG, and LDH, and chest x-ray were within normal limits. The abdominal-pelvic CT scan with contrast revealed a mass that measured 31.1 × 15.1 × 30.5 cm, with fat density (−3 to −55 HU) extended to the funiculus spermaticus, MRI was not performed because the results of the CECT examination were considered sufficiently clear and the MRI examination would not change the decision to perform exploration and excision of the tumor. The right testicle was within normal limits. There was no paraaortic, parailliac, or inguinal lymphadenopathy (Fig. 2).

**Fig. 3:**
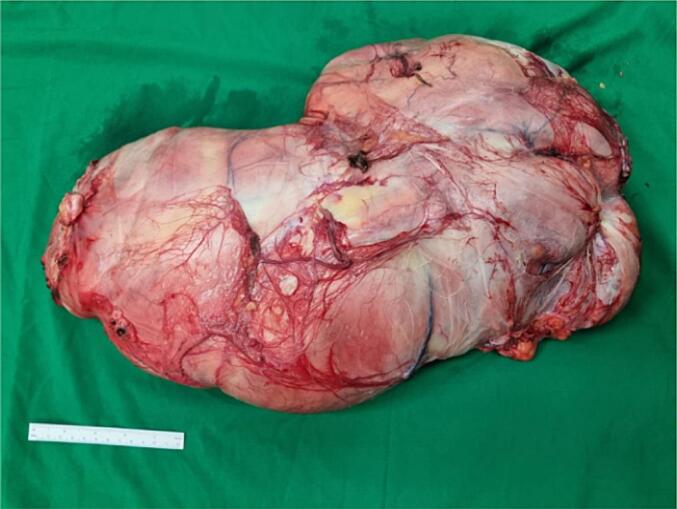
Left paratesticular tumor.

Exploration and excision of the tumor were done, with an incision from the scrotum to the left inguinal area. The tumor could be easily removed, as it was not attached to the left testicle and spermatic cord. The tumor was successfully removed without rupture, measured 39.0 × 37.0 × 16.0 cm and weighing 10 kg (Fig. 3).

**Fig. 4:**
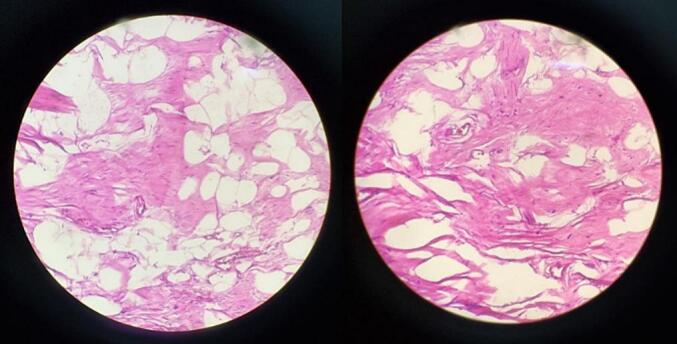
Anatomical pathology examination findings with Hematoxylin-Eosin staining.

**Fig. 5:**
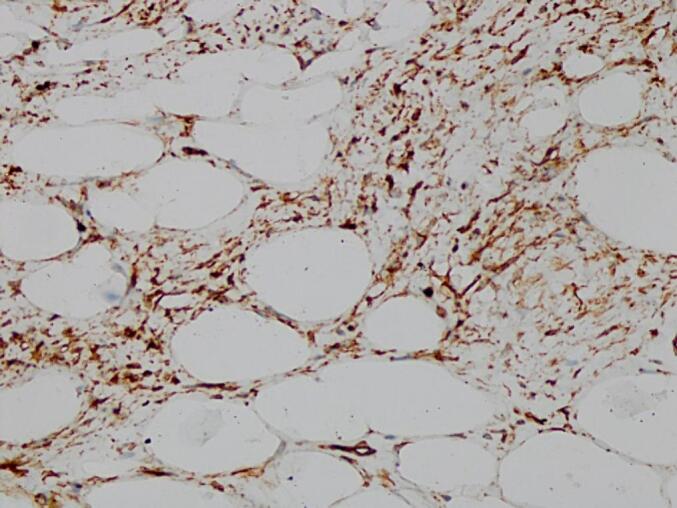
Positive results of CD34 antibody immunohistochemistry examination.

The pathology anatomy examination revealed that the tumor tissue consisted of fibro-collagen bundles and hyperplastic mature fat cells without any malignant features, aligned with the characteristics of spindle cell lipoma (Fig. 4). The diagnosis was confirmed through a CD34 immunohistochemistry examination where it showed positive results (Fig. 5). Furthermore, the objective was to exclude the presence of liposarcoma as part of the differential diagnosis.

In our case, a three-month evaluation revealed no complaints or recurrence of the mass, and the patient was able to engage in normal activities, especially urination and sexual activity.

## 3 Discussion

Spindle cell lipoma (SCL) is a rare type of lipoma that originates in the subcutaneous tissue and is characterized histopathologically by the replacement of mature adipocytes by collagenous tissue that forms spindle cells [8]. The incidence of SCL in other organs is infrequent, accounting for only 1.5 % of all cases. Meanwhile, only four cases of paratesticular SCL have been reported to date [1,[3], [4], [5]].

From several previous case reports, paratesticular SCL appeared as a painless growth of the scrotal mass, soft, sticky, and palpable on one side of the scrotum, sometimes often considered as an inguinal hernia [1,5]. This case is similar to ours, in which the patient presented with a complaint of a mass on the left side that had been known for nine years and was gradually increasing in size. The mass was described as painless and was not associated with weight loss. Physical examination revealed a palpable mass measuring 30 × 30 cm, characterized by its soft texture and smooth surface.

The nature and origin of Spindle Cell Lipoma are not yet fully understood due to the difficulty in distinguishing between early fibroblasts and prelipoblasts, even with electron microscopy [3]. The lesion of Spindle Cell Carcinoma is thought to be a dendritic interstitial neoplasm located in fat, which is not a true lipogenic neoplasm. Unlike liposarcoma, spindle cell lipoma is a benign lesion [3]. There is a progressive increase in collagen content, evaluated from the histologic appearance over the years. Like other mesenchymal tumors, its histogenesis is unclear, and the relationship of endocrine or hereditary factors that stimulate the growth of this tumor remains in question [8].

Spindle cell lipoma is difficult to distinguish radiologically from liposarcoma. Therefore, a biopsy and histopathological examination must be made to establish the diagnosis. Immunohistochemistry tests, such as CD34, can also help distinguish SCL from liposarcoma, although they are not specific [1,9]. Histopathologically, SCL has a uniform pattern of mature adipocytes and round cells interspersed with eosinophilic collagen clusters in the myxoid stroma [1,5]. In our case, histopathological examination showed tumor tissue composed of fibrous collagen bundles and hyperplastic mature adipocytes without any signs of malignancy, which matched the picture of spindle cell lipoma. In addition, CD34 immunohistochemistry tests were performed, with positive results, made the diagnosis gentle.

The management of SCL requires a total excision and is considered sufficient as therapy, without recurrence or metastasis, within six months to 5 years [3,5]. In our case, exploration and complete excision of the mass were carried out due to the huge size, with an incision in the scrotum to the left inguinal. Orchiectomy was not performed, as the mass was not found to be attached to the left testicle and funiculus spermaticus, thus it could be removed thoroughly without rupture.

SCL has a good prognosis with complete resection of the mass with or without orchiectomy, with no recurrence or metastasis at six months and five years, according to the observations of the two previous cases [3,5]. In our case, a three-month evaluation revealed no complaints or recurrence of the mass, and the patient was able to engage in normal activities, especially urination and sexual activity.

The case we encountered provides new insights into the management of Spindle Cell Lipoma, a condition with a rare incidence of all lipomas, it is even rarer to find it present in the spermatic cord, as demonstrated by a literature search revealing only a small number of case reports [1], and does not yet have a general consensus for its therapy [6].

## 4 Conclusion

Spindle cell lipoma (SCL) is a benign neoplasm. As reported in several case studies, total excision is the current optimal therapeutic approach, resulted in no recurrence. It is crucial to consider the differential diagnosis, particularly liposarcoma, known for its high recurrence rate (30–50 % within five years). The CD34 immunohistochemistry examination is essential to confirm the diagnosis, showing positive results in SCL and negative results in liposarcoma. With thorough preoperative preparation and precise post-operative diagnosis, favorable outcomes can be achieved without recurrence in cases of paratesticular SCL.

Paratesticular SCL cases are rare. This case report could contribute to further research and play a significant role to determine the appropriate diagnosis and management of paratesticular SCL cases.

## Consent

The patient provided written informed consent to publish this case report and accompanying images. A copy of the written permission is available for review by the Editor-in-Chief of this journal upon request.

## Provenance and peer review

Not commissioned, externally peer-reviewed.

## Ethical approval

Ethical approval has been acquired in this study by Health Research Ethics Committee of Dr. Soetomo General-Academic Hospital, Surabaya, Indonesia on January 14th, 2024.

## Funding

The authors received no financial support for this article's research, authorship, and publication.

## Author contribution

Putu Kurnia Darma Pratama: Conceptualization, Writing - original draft, Writing - review & editing, Investigation Lukman Hakim: Conceptualization, Resources, Supervision, Writing - original draft. Tetuka Bagus Laksita: Conceptualization, Investigation, Resources, Writing - original draft, Project administration. Karinda Triharyu Caesari Putri: Conceptualization, Investigation, Writing - original draft, Writing - review & editing.

## Guarantor

Putu Kurnia Darma Pratama.

## Research registration number

NA.

## Conflict of interest statement

The authors report no declarations of interest.
